# Analysis of Jak2 Catalytic Function by Peptide Microarrays: The Role of the JH2 Domain and V617F Mutation

**DOI:** 10.1371/journal.pone.0018522

**Published:** 2011-04-18

**Authors:** Arturo Sanz, Daniela Ungureanu, Tuija Pekkala, Rob Ruijtenbeek, Ivo P. Touw, Riet Hilhorst, Olli Silvennoinen

**Affiliations:** 1 Department of Hematology, Erasmus MC, Rotterdam, Netherlands; 2 Institute of Biomedical Technology, University of Tampere, Tampere, Finland; 3 PamGene International BV, 's-Hertogenbosch, The Netherlands; 4 Tampere University Hospital, Tampere, Finland; University of Minnesota, United States of America

## Abstract

Janus kinase 2 (JAK2) initiates signaling from several cytokine receptors and is required for biological responses such as erythropoiesis. JAK2 activity is controlled by regulatory proteins such as Suppressor of Cytokine Signaling (SOCS) proteins and protein tyrosine phosphatases. JAK2 activity is also intrinsically controlled by regulatory domains, where the pseudokinase (JAK homology 2, JH2) domain has been shown to play an essential role. The physiological role of the JH2 domain in the regulation of JAK2 activity was highlighted by the discovery of the acquired missense point mutation V617F in myeloproliferative neoplasms (MPN). Hence, determining the precise role of this domain is critical for understanding disease pathogenesis and design of new treatment modalities. Here, we have evaluated the effect of inter-domain interactions in kinase activity and substrate specificity. By using for the first time purified recombinant JAK2 proteins and a novel peptide micro-array platform, we have determined initial phosphorylation rates and peptide substrate preference for the recombinant kinase domain (JH1) of JAK2, and two constructs comprising both the kinase and pseudokinase domains (JH1-JH2) of JAK2. The data demonstrate that (i) JH2 drastically decreases the activity of the JAK2 JH1 domain, (ii) JH2 increased the K_m_ for ATP (iii) JH2 modulates the peptide preference of JAK2 (iv) the V617F mutation partially releases this inhibitory mechanism but does not significantly affect substrate preference or K_m_ for ATP. These results provide the biochemical basis for understanding the interaction between the kinase and the pseudokinase domain of JAK2 and identify a novel regulatory role for the JAK2 pseudokinase domain. Additionally, this method can be used to identify new regulatory mechanisms for protein kinases that provide a better platform for designing specific strategies for therapeutic approaches.

## Introduction

Most cytokine receptors lack intrinsic kinase activity and rely on Janus kinases (JAKs) for signaling [1], [2]. Members of the JAK family of kinases have a characteristic domain organization consisting of seven JAK homology domains (JH domains 1–7). The N-terminal segment (JH7-JH3 domains) contains a FERM (band 4.1 ezrin, radixin and moesin) domain as well as an atypical SH2 (Src-homology-2 domain)-like domain which have been shown to mediate association with the membrane-proximal region of cytokine receptors [1]. The C-terminus of these proteins comprises the JH1 domain that contains classical motifs required for kinase catalysis and functions as the catalytic site. The JH2 domain, located between the SH2-like and the JH1 domains, has been predicted to be catalytically inactive due to the lack of essential amino-acids in the catalytic consensus motifs of kinases and has been classified as a pseudokinase domain [3]. However, the JH2 domain has been shown to have an important regulatory role in JAK activation [4], [5].

Clinical evidence for the relevance of this domain was obtained in 2005, when an acquired point mutation in the JH2 domain of JAK2 (Val 617 to Phe substitution, V617F) was identified in myeloproliferative neoplasm (MPN) patients [6], [7], [8], [9]. This mutation resulted in increased JAK2-mediated signaling and conferred the MPN phenotype in a mouse bone marrow transplantation model [10], [11], [12]. These findings intensified the analysis of disease-associated mutations in JAKs and led to identification of several mutations in JAK1, JAK2, JAK3 and Tyk2 in different diseases. Interestingly, although these mutations are found in all JH domains, the majority of them affect the pseudokinase (JH2) domain [13], [14], further supporting the critical role of this domain in human JAK signaling.

The underlying mechanisms of the JH2 mediated regulation have remained largely unknown. The difficulties in producing and purifying JH2 domains have hampered analysis of their effect on JAK2 enzymology, function and signaling. Nonetheless, although structural evidence is still lacking, biochemical evidence suggests that this regulation is mediated through intramolecular interactions between the JH1 and JH2 domains [15].

Among the essential characteristics of any given enzyme, its ability to recognize the appropriate substrate is critical, as this ensures cellular signals to be transmitted correctly. Previously, it was shown that tyrosine kinases and Src-homology-2 (SH2) domains recognize amino acid residues in a specific sequence context that provides specificity to signal transduction [16], [17], [18]. Moreover, examples that this specificity can be altered by single point mutations have been described [19]. Changes in specificity may cause phosphorylation and activation of unpredicted targets that lead to disease states. Understanding the functional consequences of these changes may contribute to a more effective designing of selective and clinically valuable drugs to inhibit the activity of mutant kinases.

In this study, we have overcome previous difficulties in producing and purifying sufficient quantities of protein for detailed evaluation of these domains by using a baculovirus system. In addition, we have expanded the application of a recently developed multiplex array technology [20], [21], [22], [23], [24], [25], to study the effect of inter-domain interactions in kinase activity and substrate preference. Our results demonstrate the role of the JH2 domain as a specific intramolecular modulator of JAK2 kinase. This domain modulates peptide preference and profoundly regulates the activity of JAK2 enzyme. In addition, we found that mutation V617F partially relieves the inhibition of the JH2 domain on catalytic activity but has limited effect on substrate preference.

## Results

### Protein purification and substrate identification

**Figure 1:**
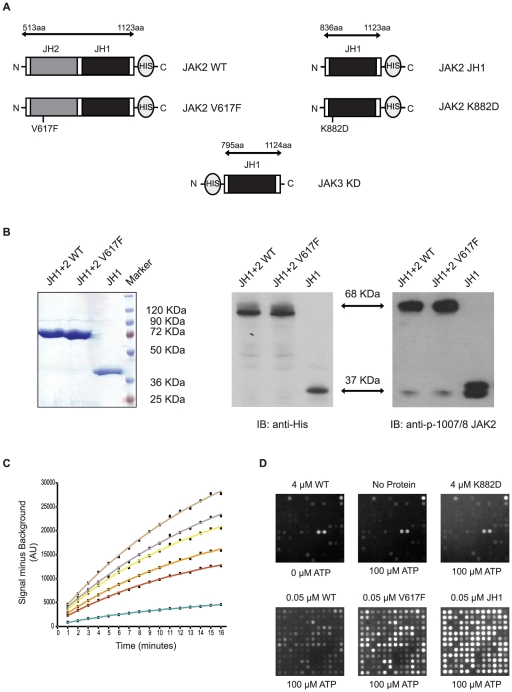
Protein characterization. **A**. Schematic representation of the proteins encoded by the different constructs. Amino acid boundaries for each construct are indicated, numbers refer to the human JAK2 sequence. A HIS tag is located in the C or N terminus according to the figure and indicated by the suffix HIS in the text. **B**. JAK2 JH1-JH2WT and V617F mutant along with JH1 domain were produced in Sf9 cells and purified by Ni-NTA affinity and gel-size chromatography. Purified proteins were concentrated to 1 mg/ml (as shown by Coomassie staining, left), immunoblotted with anti-HIS (middle) and anti-pTyr1007/1008 JAK2 (right) antibodies. **C**. Example of a time-dependent reaction progress curve for JAK2 JH1 at different concentrations of STAT5A_687_699 peptide: 100 µM, Green; 300 µM, Red; 400 µM, Orange; 600 µM, Yellow; 750 µM, Grey and 1000 µM, Brown. Initial rates (v) for each peptide on the array were obtained by fitting the data points to the equation for exponential association, as described in Materials and Methods. **D**. Images of PamChip® peptide microarrays comprising 144 Tyr containing peptides. Images were taken after 30 min of incubation with complete JAK assay mix.

HIS-tagged JH1-JH2WT, JH1-JH2V617F and JH1 domains from JAK2 (Figure 1A) were produced in Sf9 cells using a Bac-to-Bac expression system and were purified using a two-step purification protocol. JAK2 proteins were first purified from infected Sf9 cell pellets using Ni-NTA affinity beads, followed by size-exclusion chromatography. Fractions containing JAK2 proteins were eluted as monomers and concentrated for further analysis. As shown by Coomassie staining in Figure 1B, all proteins showed high purity. Phosphorylation of the activation loop of JAK2, as with other protein tyrosine kinases, is essential for its activation [26]. In consequence, the phosphorylation state of Tyr1007-Tyr1008 in the activation loop was monitored by Western blot using a specific anti-JAK2 p-Tyr antibody (Figure 1B). For all JAK proteins, the activation loop tyrosines were phosphorylated and minor differences were found in the level of phosphorylation between the wild type and the mutant form.

Peptide microarrays provide a high content platform to identify substrates and determine kinetic parameters for protein kinases in a highly multiplex fashion [24], [25]. As an initial screening for substrate identification the different JAK2 proteins were incubated on PamChip® 96 microarray plates. Each array contains 144 peptide sequences derived from putative tyrosine-phosphorylation sites in human proteins. Assay conditions have been optimized with respect to amount of antibody, to assure that the initial reaction rate reflects the rate of enzymatic conversion and not antibody binding. The detection antibody has been reported to have a broad specificity for phosphopetides [27]. An example of the time-dependent progression of the reaction at different concentrations of peptide is given in Figure 1C. Initial velocities (v) were obtained by fitting the data points of the time series to the equation for exponential association and calculating the tangent at 2 minutes, as indicated in Materials and Methods. Arrays were incubated with a fixed concentration of ATP (100 µM) and increasing concentrations of protein (ranging from 0 to 8 pmoles per reaction) to assure that phosphorylation rates increase linearly with protein concentration. Similarly, a fixed concentration of protein was incubated with increasing concentrations of ATP (ranging from 0 to 400 µM). Peptide phosphorylation was monitored in real time by taking images with an integrated CCD-based optical system and data were analyzed as described in Experimental procedures. Representative images of the arrays after 30 minutes are shown in Figure 1D, along with images of negative controls. Nonspecific signals were identified by performing incubations without protein or ATP (Figure 1D) and by inhibiting the reaction with 10 µM Staurosporine or 100 µM AMP-PNP. Incubation with the catalytically inactive K882D JAK2 kinase was also used as negative control (Figure 1D). Furthermore, occurrence of potential artifacts due to binding of autophosphorylated proteins to non-phosphorylated peptides was monitored by pre-incubating proteins with ATP (400 µM) for 30 minutes and stopping the reaction with 10 µM Staurosporine before incubation on a PamChip® (data not shown). Nonspecific signals were not considered for further analysis. Substrates were then defined as those peptides having signal intensities higher than two times the standard deviation of the background after 20 minutes incubation and their signals must increase with enzyme and ATP concentration.

This initial screening showed that JH1 phosphorylated 63 out of 144 peptides above background level on the array, while JH1-JH2WT and JH1-JH2V617F phosphorylated significantly fewer peptides, 27 and 42 peptides, respectively. All peptides that were phosphorylated by JH1-JH2WT and JH1-JH2V617F were also phosphorylated by JH1, indicating that JH2 domain limits but does not drastically change the phosphorylation specificity pattern of the JAK2 tyrosine kinase domain. The list of phosphorylated peptides is given in Table 1. These results were then used to produce custom PamChip® microarrays, as indicated in the Materials and Methods section.

**Table 1:** Substrate identification by PamChip® 144 peptide microarray.

Protein Description	Peptide ID	Peptide Sequence	WT	V617F	JH1
Protein 4.1	41_654_666	LDGENIYIRHSNL			X
Acetylcholine receptor subunit delta precursor	ACHD_383_395	YISKAEEYFLLKS	X	X	X
Band 3 anion transport protein	B3AT_39_51	TEATATDYHTTSH		X	X
Complement C1r subcomponent precursor	C1R_199_211	TEASGYISSLEYP		X	X
Calmodulin	CALM_93_105	FDKDGNGYISAAE		X	X
	CALM_95_107	KDGNGYISAAELR		X	X
E3 ubiquitin-protein ligase CBL	CBL_693_705	EGEEDTEYMTPSS			X
Proto-oncogene C-crk	CRK_214_226	GPPEPGPYAQPSV		X	X
Catenin beta-1	CTNB1_79_91	VADIDGQYAMTRA			X
Dual specificity tyrosine-phosphorylation-regulated kinase 1A	DYR1A_212_224	KHDTEMKYYIVHL			X
Embryonal Fyn-associated substrate	EFS_246_258	GGTDEGIYDVPLL			X
Epidermal growth factor receptor precursor	EGFR_1103_1115	GSVQNPVYHNQPL			X
	EGFR_1165_1177	ISLDNPDYQQDFF		X	X
	EGFR_1190_1202	STAENAEYLRVAP	X	X	X
Ephrin type-A receptor 2 precursor	EPHA2_765_777	EDDPEATYTTSGG			X
Ephrin type-A receptor 4 precursor	EPHA4_589_601	LNQGVRTYVDPFT		X	X
Ephrin type-B receptor 1 precursor	EPHB1_771_783	DDTSDPTYTSSLG			X
Erythropoietin receptor precursor	EPOR_361_373	SEHAQDTYLVLDK	X	X	X
	EPOR_419_431	ASAASFEYTILDP	X	X	X
Receptor Tyrosine-protein kinase erbB-2 precursor	ERBB2_1241_1253	PTAENPEYLGLDV	X	X	X
Receptor tyrosine-protein kinase erbB-4 precursor	ERBB4_1181_1193	QALDNPEYHNASN	X	X	X
	ERBB4_1277_1289	IVAENPEYLSEFS	X	X	X
Fatty acid-binding protein, heart	FABPH_13_25	DSKNFDDYMKSLG			X
Focal adhesion kinase 1	FAK1_569_581	RYMEDSTYYKASK		X	X
Protein tyrosine kinase 2 beta	FAK2_572_584	RYIEDEDYYKASV		X	X
Proto-oncogene tyrosine-protein kinase FER	FER_707_719	RQEDGGVYSSSGL			X
Fibroblast growth factor receptor 1 precursor	FGFR1_761_773	TSNQEYLDLSMPL	X	X	X
Fibroblast growth factor receptor 2 precursor	FGFR2_762_774	TLTTNEEYLDLSQ	X	X	X
Fibroblast growth factor receptor 3 precursor	FGFR3_641_653	DVHNLDYYKKTTN		X	X
	FGFR3_753_765	TVTSTDEYLDLSA	X	X	X
Insulin receptor precursor	INSR_992_1004	YASSNPEYLSASD	X	X	X
Tyrosine-protein kinase JAK1	JAK1_1015_1027	AIETDKEYYTVKD	X	X	X
Tyrosine-protein kinase JAK2	JAK2_563_577	VRREVGDYGQLHETE		X	X
Tyrosine-protein kinase SYK	KSYK_518_530	ALRADENYYKAQT	X	X	X
Linker for activation of T-cells family member 1	LAT_194_206	MESIDDYVNVPES	X	X	X
Mitogen-activated protein kinase 7	MK07_211_223	AEHQYFMTEYVAT	X	X	X
Mitogen-activated protein kinase 12	MK12_178_190	ADSEMTGYVVTRW	X	X	X
Mitogen-activated protein kinase 14	MK14_173_185	RHTDDEMTGYVAT	X	X	X
BDNF/NT-3 growth factors receptor precursor	NTRK2_509_521	PVIENPQYFGITN			X
	NTRK2_696_708	GMSRDVYSTDYYR	X	X	X
Paxillin	PAXI_111_123	VGEEEHVYSFPNK			X
	PAXI_24_36	FLSEETPYSYPTG			X
Beta-type platelet-derived growth factor receptor	PGFRB_1002_1014	LDTSSVLYTAVQP			X
precursor	PGFRB_1014_1028	PNEGDNDYIIPLPDP	X	X	X
	PGFRB_572_584	VSSDGHEYIYVDP		X	X
	PGFRB_709_721	RPPSAELYSNALP			X
	PGFRB_768_780	SSNYMAPYDNYVP			X
	PGFRB_771_783	YMAPYDNYVPSAP			X
Serine/threonine-protein phosphatase 2A catalytic beta	PP2AB_297_309	EPHVTRRTPDYFL	X	X	X
Paired mesoderm homeobox protein 2	PRRX2_202_214	WTASSPYSTVPPY			X
Macrophage-stimulating protein receptor precursor	RON_1346_1358	SALLGDHYVQLPA	X	X	X
	RON_1353_1365	YVQLPATYMNLGP	X	X	X
Signal transducer and activator of transcription 5A	STA5A_687_699	LAKAVDGYVKPQI	X	X	X
Signal transducer and activator of transcription 1-alpha/beta	STAT1_694_706	DGPKGTGYIKTEL			X
Signal transducer and activator of transcription 3	STAT3_698_710	DPGSAAPYLKTKF		X	X
Signal transducer and activator of transcription 4	STAT4_686_698	TERGDKGYVPSVF			X
Vascular endothelial growth factor receptor 1	VGFR1_1162_1174	VQQDGKDYIPINA		X	X
precursor	VGFR1_1320_1332	SSSPPPDYNSVVL	X	X	X
	VGFR1_1326_1338	DYNSVVLYSTPPI	X	X	X
	VGFR2_1052_1064	DIYKDPDYVRKGD			X
	VGFR2_1168_1180	AQQDGKDYIVLPI	X	X	X
Vinculin	VINC_815_827	KSFLDSGYRILGA	X	X	X
Tyrosine-protein kinase ZAP-70	ZAP70_485_497	ALGADDSYYTARS	X	X	X

Peptide ID is based on the UniProt Knowledgebase, and the numbers indicate the position of the first and last amino acids of the peptide in the complete human protein (UniProt annotation and numbering). Substrates were defined as those peptides showing protein- and ATP-concentration dependent signals after incubation. Nonspecific signals were not considered. In the three columns on the right hand side of the table, an X indicates phosphorylation of that peptide by the JAK2 construct indicated in the heading.

### Activity of the proteins

**Figure 2:**
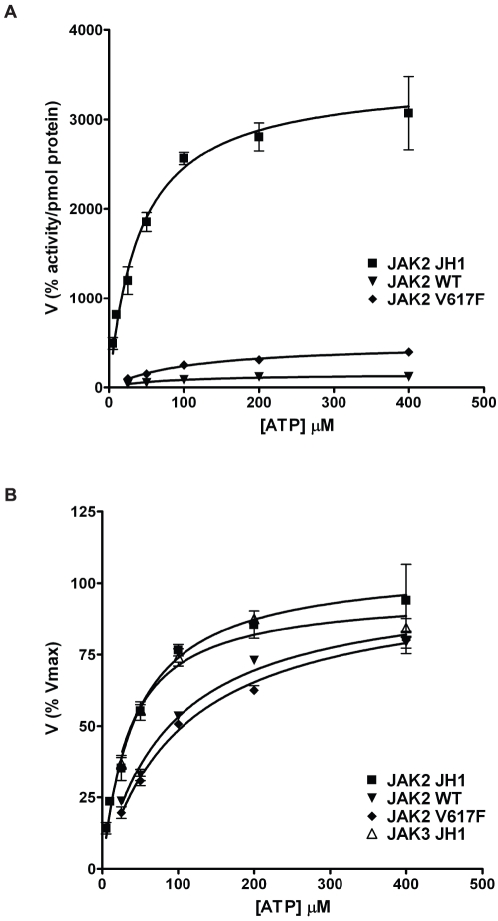
Contribution of the different domains to the catalytic activity of JAK2 JH1. **A**. Comparison of the activity per pmol protein for JAK2 JH1, JAK2 JH1-JH2WT and JH1-JH2V617F acting on 1000 µM STAT5A_687_699 as a function of ATP concentration. The activity for each protein was calculated in relation to the initial rates obtained at 100 µM ATP and expressed per pmol of protein used. Values are the average of 3 v-values. **B**. Comparison of relative activity for JAK2 JH1, JAK3 JH1, JAK2 JH1-JH2WT and JH1-JH2V617F. Relative activity was calculated in relation to the V_max_ for each protein. Values are the average of 3 v-values.

Previous studies have shown that JH1 is more active than JH1-JH2 leading to the conclusion that JH2 regulates the activity of JH1 [4], [5]. Therefore, we compared the activity of HIS-tagged JH1 and JH1-JH2 proteins on custom PamChip® microarrays. In an initial experiment, the amount of protein was optimized as indicated above. Subsequently, phosphorylation rates on 1000 µM STAT5A_687_699 were determined. Relative activity for each protein was obtained in relation to the initial rates obtained at 100 µM ATP and corrected for the amount of protein used. Figure 2A shows the activity for the three JAK2 proteins per pmol of enzyme. JAK2 JH1-JH2WT was clearly the least active protein. When compared to JH1-JH2WT (V_max_, _app_ = 150±9 relative activity per pmol protein), mutation on V617F (V_max_, _app_ = 493±19 relative activity per pmol protein) produced a three fold increase, whereas the absence of the JH2 domain resulted in a 20 fold increase in activity for the JH1 form (V_max_, _app_ = 3467±180.7 relative activity per pmol protein).

### Comparison of Michaelis constants (K_m_) for ATP in JAK kinase domains

All kinases catalyze a two-substrate reaction between ATP and a phosphate acceptor. Therefore, the rate of the reaction is determined by the concentration of both substrates. Accurate determination of peptide substrate preference requires a concentration of ATP at or near saturation in order to avoid ATP to be a limiting factor for the reaction. Comparison of the normalized activities for phosphorylation of 1000 µM STAT5A_687_699 at different concentrations of ATP is presented in Figure 2B. The JAK3 JH1 protein (containing only the JH1 domain of JAK3) was included for substrate comparison, and the K_m_ values for ATP were also determined for this protein. The analysis showed that proteins containing both JH1 and JH2 presented higher K_m_ values for ATP (WT = 88±9 µM and V617F = 106±11 µM) than proteins containing the kinase domain alone (JAK2 JH1 = 44±6 µM and JAK3 JH1 = 35±5 µM). Similar differences in K_m_ values were found when other peptides were used as substrates (data not shown). The K_m_ values for the kinase domains of JAK2 and JAK3 with the STAT5A peptide are slightly higher than those obtained in a recent study [28]. Taken together, our data shows that the JH2 domain reduces the affinity for ATP and that the V617F mutation has little or no effect on this parameter.

### Catalytic efficiency of JAK2 kinase domain

The effect of the peptide sequence on the kinase reaction can be studied by determining enzymological parameters such as the Michaelis-Menten constant (K_m_) and the maximal velocity (V_max_) per peptide. However, in most cases, enzymes show the largest differences in the V_max_ rather than in K_m_ for different substrates. This is because substrate specificity often results from differences in transition state, rather than ground state binding interaction [29]. In addition, reliable V_max_ and K_m_ values can only be calculated when the substrate concentrations [S] are several times higher than K_m_, a condition that is difficult to meet for peptides that are poor substrates (Figure S2). Consequently, the most general way to compare different substrates is the catalytic efficiency (V_max_/K_m_), which also reflects the ratio of conversion when a mixture of substrates is offered to an enzyme. When [S]<<K_m_ the relation of a plot v (initial velocity) versus [S] is linear and the V_max_/K_m_ can be obtained from the slope of this plot [29]. Therefore, V_max_/K_m_ values were calculated from the linear part of v vs. [peptide] plots. The percentage of activity in relation to 1000 µM STA5A_687_699, expressed per pmol of protein was used to obtain V_max_/K_m_ values. In order to prevent limiting concentrations of ATP, V_max_/K_m_ values for the different proteins were obtained at 400 µM ATP and are shown in Table 2. To facilitate comparison of these values, two transformations of these results were performed. First, JH1-JH2WT was set to 1 for all substrates and the fold change with respect to the other proteins was calculated. This transformation levels off the catalytic efficiency specific for each peptide, while maintaining differences in both activity and specificity of the different proteins. Secondly, V_max_/K_m_ values for each JAK2 form were expressed as the percentage of the sum of all V_max_/K_m_. This transformation averages the differences in activities, while maintaining differences in peptide specificity.

**Table 2:** Catalytic efficiency (V_max_/K_m_) at 400 µM ATP.

		JAK2	WT			JAK2	V617F			JAK2	JH1			JAK3	JH1	
Peptide ID	Vmax/Km	R^2^	Fold Change	% total Vmax/Km	Vmax/Km	R^2^	Fold Change	% total Vmax/Km	Vmax/Km	R^2^	Fold Change	% total Vmax/Km	Vmax/Km	R^2^	Fold Change	% total Vmax/Km
**EGFR_1103_1115**	0.016±0.008	0,378	1	1	0.061±0.017	0,711	4	1	0.345±0.140	0,670	22	1	0.073±0.003	0,968	5	1
**EGFR_1165_1177**	0.062±0.006	0,889	1	2	0.159±0.006	0,981	3	2	0.880±0.075	0,895	14	2	0.474±0.025	0,959	8	6
**EGFR_1190_1202**	0.314±0.020	0,941	1	11	0.956±0.041	0,971	3	10	2.928±0.311	0,872	9	6	0.330±0.024	0,921	1	4
**EPOR_361_373**	0.079±0.009	0,822	1	3	0.320±0.019	0,946	4	3	2.560±0.268	0,851	32	5	0.122±0.007	0,946	2	2
**EPOR_419_431**	0.129±0.006	0,967	1	5	0.366±0.021	0,948	3	4	2.471±0.241	0,868	19	5	0.210±0.009	0,972	2	3
**ERBB2_1241_1253**	0.241±0.017	0,923	1	9	0.703±0.053	0,918	3	8	2.905±0.350	0,852	12	6	0.304±0.025	0,899	1	4
**FAK1_569_581**	0.044±0.004	0,897	1	2	0.163±0.006	0,980	4	2	0.735±0.064	0,890	17	1	0.199±0.014	0,921	4	3
**FAK2_572_584**	0.143±0.010	0,927	1	5	0.441±0.012	0,987	3	5	3.456±0.128	0,984	24	7	0.631±0.033	0,959	4	8
**INSR_992_1004**	0.055±0.005	0,893	1	2	0.136±0.011	0,903	2	1	1.252±0.088	0,939	23	2	0.330±0.010	0,985	6	4
**JAK1_1015_1027**	0.143±0.008	0,957	1	5	0.446±0.026	0,946	3	5	3.623±0.248	0,947	25	7	0.643±0.034	0,957	4	8
**JAK2_563_577**	0.053±0.005	0,894	1	2	0.126±0.008	0,935	2	1	0.889±0.052	0,949	17	2	0.107±0.005	0,960	2	1
**PGFRB_1002_1014**	0.032±0.004	0,826	1	1	0.130±0.006	0,971	4	1	0.879±0.051	0,948	27	2	0.069±0.005	0,909	2	1
**PGFRB_1014_1028**	0.231±0.015	0,937	1	8	0.784±0.025	0,984	3	8	3.946±0.561	0,818	17	8	0.433±0.022	0,962	2	6
**PGFRB_572_584**	0.375±0.016	0,972	1	13	1.090±0.060	0,953	3	12	3.750±0.369	0,888	10	7	0.818±0.082	0,861	2	11
**PGFRB_709_721**	0.022±0.002	0,887	1	1	0.078±0.005	0,942	4	1	0.433±0.045	0,850	20	1	0.065±0.006	0,883	3	1
**PGFRB_768_780**	0.027±0.003	0,867	1	1	0.083±0.006	0,917	3	1	0.500±0.037	0,920	18	1	0.191±0.011	0,950	7	3
**PGFRB_771_783**	0.043±0.004	0,894	1	2	0.135±0.005	0,980	3	1	0.908±0.070	0,913	21	2	0.220±0.006	0,989	5	3
**PP2AB_297_309**	0.091±0.004	0,973	1	3	0.335±0.014	0,972	4	4	1.626±0.079	0,964	18	3	0.779±0.039	0,962	9	10
**RON_1346_1358**	0.197±0.019	0,901	1	7	0.675±0.035	0,959	3	7	7.388±0.803	0,914	38	15	0.408±0.021	0,961	2	5
**RON_1353_1365**	0.148±0.009	0,947	1	5	0.762±0.021	0,988	5	8	3.241±0.260	0,906	22	6	0.313±0.012	0,978	2	4
**STA5A_687_699**	0.106±0.007	0,946	1	4	0.425±0.017	0,974	4	5	2.087±0.129	0,942	20	4	0.073±0.004	0,957	1	1
**STAT1_694_706**	0.033±0.004	0,843	1	1	0.149±0.007	0,965	4	2	0.860±0.054	0,940	26	2	0.034±0.004	0,847	1	0
**STAT3_698_710**	0.066±0.005	0,927	1	2	0.255±0.008	0,984	4	3	0.819±0.069	0,933	12	2	0.071±0.003	0,979	1	1
**STAT4_686_698**	0.027±0.003	0,846	1	1	0.116±0.009	0,911	4	1	0.505±0.035	0,928	19	1	0.091±0.007	0,904	3	1

V_max_/K_m_ values were calculated from the linear part of v vs. [peptide] plots generated on a custom PamChip® peptide array. The fold change for each peptide with respect to JH1-JH2 WT and the percentage of the sum of all V_max_/K_m_ for each protein are also indicated.

Most kinases show highly similar structures, yet phosphorylate different substrates. Kinase and substrate interact on basis of charge, hydrogen bonding or hydrophobic interactions that make the substrate fit perfectly in the active site [30]. Therefore, the largest differences in substrate preferences should appear between distinct catalytic cores, as distinct interactions may take place. A comparative experiment was performed with JAK3 JH1 to determine the ability of this method to detect these differences. As shown in Table 2, the catalytic efficiency for both JAK2 JH1 and JAK3 JH1 significantly varies for some substrates while remaining similar for others. These results validate our methodology and support that, although JAK2 and JAK3 show 70% identity between their crystal structures, these differences are sufficient to robustly alter peptide preference [31], [32]. Presumably, the presence of additional regulatory domains may also affect substrate preference, as interactions may also change. The values obtained for JAK2 JH1 (Table 2) reflect this effect, as the fold change varies over wide ranges when compare to WT (8 to almost 40-fold) and significant differences are also found in the percentage of the total catalytic efficiency for certain peptides. On the contrary, the catalytic efficiency for JAK2 V617F increases 2–5 folds as compared to the WT and catalytic efficiencies, expressed as percentage of the total, appeared similar for both JH2 containing constructs. This suggests that the V617F mutation only increases the activity, but has little or no effect on substrate preference.

### JAK2 substrate motif

**Figure 3:**
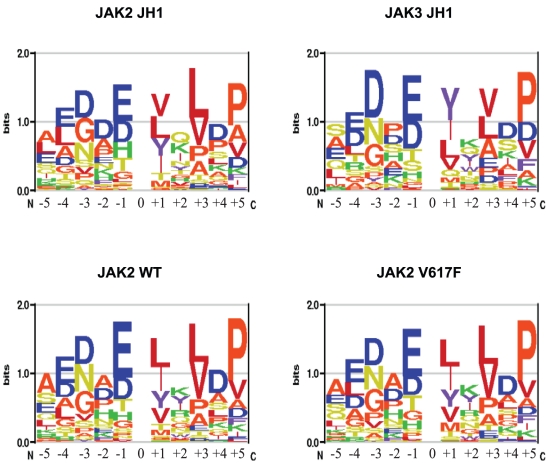
Sequence recognition motifs. The motif was obtained by expressing V_max_/K_m_ for each peptide as a fraction of the total V_max_/K_m_ obtained on the chip and entering the weighted contribution into the enoLOGOS program. The height of the stack of single amino acid letters indicates the relative entropy of the site. The size of each letter indicates its preference at the position relative to the phosphorylation site between −5 and +5.

To better understand the determinants of substrate recognition, we investigated whether comparison of the primary sequence of the substrate peptides could reveal a differential consensus motif for the different proteins. For this purpose, the peptide sequences from Table 2 were aligned relative to the central Tyr residue and only residues from position −5 to +5 were considered. For each protein, only the percentage of the total V_max_/K_m_ for each peptide was entered as weighted sequences into the enoLOGOS program [33] and the result for each form is presented in Figure 3.

In general, all constructs preferred peptide sequences with negatively charged amino acid at the N-terminus and hydrophobic residues at the C-terminus of the Tyr residue. In particular, the presence of Gly, Glu or Asn at position −3, Glu or Asp at position −1, Leu and/or Val at positions +1 and +3 and Pro at +5 seems to favor substrate phosphorylation. Interestingly, peptides with two adjacent tyrosine residues, like Tyr1007 and Tyr1008 in the activation loop of JAK2 [26], were also found to be preferred. This is in agreement with current knowledge suggesting that most protein tyrosine kinases (PTKs) prefer Asp and Glu at the N-terminus of the tyrosine residue and that strong preferences are found at positions +1 and +3 [34]. In addition, previous studies on the autophosphorylation sites of JAK2 also identified sequences with high similarities with this motif [26], [35], [36], [37]. An additional online search on PhosphoSitePlus [38] rendered several cytoplasmic proteins (like Akt, Crk, Pten, PKC, Ron, SHP, and STAT5) and membrane receptors (like CSF2RB, EPOR and PrlR) involved in JAK2 signaling with phosphorylated tyrosines in sites strongly similar to this motif. More importantly, the preferred sequence of the amino acids differs among the different proteins, as a consequence of the differences in peptide preference. This is particularly evident at positions −3, −1, +1 and +3 of the central Tyr. The largest differences are found for JAK3 JH1, which may reflect sequential and structural differences as mentioned above. The JAK2 forms, on the contrary, show more similarities among them. In this line, only small differences in substrate motifs were found between JH1-JH2WT and JH1-JH2V617F while the shift in the amino acid preference at positions −3 and +1 in the absence of the JH2 also supports the modulatory role of this domain in peptide recognition. These results provide evidence for the relevance of the amino acid sequence in the kinase-substrate interaction. In addition, subtle changes in these interactions can be detected by this method, hence providing a powerful platform for kinase analysis and drug screening.

## Discussion

After the discovery of the JAK2 V617F mutation in MPN patients, several other mutations in the JH2 domain of JAKs have been identified in MPN, but also in lymphoid and myeloid leukemia and multiple myeloma patients [14], [39]. Consequently, increasing efforts are being made to generate novel protein-tyrosine kinase inhibitors specifically targeting these mutant forms. A detailed understanding of the mechanisms of enzyme regulation is essential for the design of highly specific and highly potent modulators for the treatment of these patients with minimal side effects. Array-based platforms, as used in this study, provide useful tools for identifying such molecules. To date, the mechanisms of regulation for JAK proteins are still incompletely understood. We used peptide microarrays to study the effect of the JH1-JH2 interaction on initial reaction rates and compare catalytic efficiencies for 24 peptides. We showed that the presence of the JH2 domain not only affects kinase activity but also modulates substrate specificity of JAK2. However, mutation V617F, while inducing hyperactivity of JAK2, did not give rise to detectable changes in substrate specificity. Finally, a peptide recognition motif for JAK2 was identified that could be used for the identification of new substrates and interaction partners of JAK2.

Phosphorylation of the activation loop is essential for both mutant and wild type JAK2 [26], [40]. The conformational change induced by phosphorylation affects the catalytic activity by >50 fold [28]. Yet again, at similar phosphorylation levels, we show that the mutant form partially releases the inhibition by the JH2 domain. This is in line with previous evidence suggesting that mutation V617F leads to a hyperactive kinase [40] and explains the further enhancement in activity upon cytokine stimulation [7], [9].

The molecular models of JAK2 suggests that the JH1 and JH2 domains form an interface in an anti-symmetrical way [41], [42], [43], [44]. This interface is formed between the activation loop of JH1 and a loop between β2 and β3 strands in the N-terminal lobe of JH2, where Val 617 is localized. The interaction between JH1 and JH2 is predicted to stabilize the activation loop of JH1. In kinases, the activation loop also forms part of the peptide substrate binding pocket [45], [46] and it may, therefore, regulate substrate binding. Given that kinase and substrate have complementary sequences in a 3D space, stabilization of the activation loop in a certain position may modulate substrate preference by shifting the interactions of the complementary sequences. The analysis of multiple substrates presented in this study supports this modulatory role of the JH2 domain in peptide preference. However, very little differences in substrate preference were found when comparing both JH2 containing forms, suggesting that the effect of the amino acid change merely elevates kinase activity. Surprisingly, peptides derived from STAT proteins did not emerge as the best substrates for JAKs. One possibility is that, although peptide sequences determine the first level of substrate specificity [30], structural determinants essential for optimal substrate recognition are missing in the sequences used here. In addition, it is possible that distal sites in both the kinase and the target protein, and scaffolding proteins, elevate reaction rates by drastically increasing local concentration of the substrate [30].

The 2-fold increase in the K_m_ for ATP found in both JH2-containing proteins is of interest, because it may suggest that the JH2 domain interferes with ATP binding to the catalytically active JH1 domain. This effect could either be due to steric hindrance, or a conformational change affecting the affinity for ATP. Steric hindrance could fit the current model as the proposed JH1-JH2 interactions may physically hinder the access of ATP to the kinase domain and increase the amount of ATP required for the reaction to occur. However, this mechanism appears less likely, because steric hindrance would be overcome at high concentrations of ATP. Thus, the results support a model in which the increase in the K_m_ in JH2-containing proteins is due to a conformational change. It should, however, be noted that recently some proteins with a pseudokinase-like sequence have been demonstrated to bind ATP and display an active conformation [47], or even possess catalytic activity [48], [49], [50]. This possibility may provide additional reaction steps in the mechanism of action of JH2-containing proteins. Given that most small-molecule PTK inhibitors identified through screening of compound libraries are invariably competitive against ATP and not substrate protein/peptide, these results also suggest that development of JAK2 inhibitors based on the structure of the JH1 domain will rarely be as effective in vivo and that strategies incorporating the JH2 domain may appear more promising.

A potential limitation of the current study might be that the recombinant JAK proteins studied lacked the FERM and SH2 domains, as there is evidence that these domains, involved in receptor interaction, might also exert auto-inhibitory activities [1], [36], [51], [52], [53], [54], [55]. However, Funakoshi-Tago [56] have shown that in the absence of receptor protein, the unbound JAKs adopt a conformation that prevents the catalytic domain from being fully active. Because it is only by interaction with receptors that displacement of the FERM domain gives rise to the fully active conformation, proteins lacking the restrictive FERM domain appear more suitable for studying JAK activities in the absence of receptor proteins.

In conclusion, our studies have identified a role of the JAK2-JH2 domain in substrate preference in addition to its negative regulatory action on kinase activity mediated by the JH1 domain. The V617F mutation in the JH2 domain commonly detected in MPN increased kinase activity but did not change substrate preference. Mutations in JAKs and chimeric JAK proteins resulting from chromosomal translocations are found in different forms of acute leukemia, in particular acute lymphoblastic leukemia in Down syndrome patients and acute megakaryoblastic leukemia [14]. The methodology described in this paper will provide the opportunity to study to what extent these abnormalities give rise to changes in substrate preference or even specificity and how this might contribute to the pathogenesis of these distinct leukemia subtypes.

## Materials and Methods

### Plasmid constructs, cell lines and reagents

JAK2 (EC-2.7.10.2) proteins JH1-JH2WT, JH1-JH2V617F and JH1 were cloned into pFASTBAC1 vector (Invitrogen) with a C-terminal thrombin cleavable 6XHIS tag. The amino acids encoded by the Jak2 constructs are shown in Figure 1A and the numbering refers to human Jak2 (GenBankTM accession number NM_004972.3). Mutations were made by QuikChange site-directed mutagenesis kit (Stratagene) and verified by sequencing. Ni-NTA affinity beads for protein purification were from Qiagen and Superdex 75 gel filtration column was obtained from GE Healthcare. HIS tagged JAK3 JH1 kinase was obtained from Carna Biosciences (GenBankTM accession number NP_000206.2). Antibodies were from the following sources: anti-pTyr1007/1008 JAK2 antibodies, Cell Signaling Technology; anti-His antibody, Sigma-Aldrich; fluorescein-labeled PY20 antibody, Exalpha; and secondary biotinylated anti-Mouse or anti-Rabbit antibodies, Dako-Denmark. Streptavidin-biotinylated horseradish peroxidase complex antibody was from GE Healthcare. PamChip® Tyrosine kinase microarrays and BioNavigator software for analysis of peptide microarrays were obtained from PamGene International BV. 10× PK kinase buffer and 100× BSA for kinase assays were obtained from New England Biolabs while ATP was obtained from Sigma. Staurosporine and AMP-PNP were obtained from BioMol and Roche respectively. Prism 4 software was obtained from GraphPad Software.

### Protein Expression, Purification and Western Blots

Sf9 cells were infected with recombinant bacmid DNA containing JAK2 domains at cell density of 1×10^6^ cells/mL for virus amplification and at 2×10^6^ cells/mL for protein production. Cells were lysed in buffer containing 20 mM TRIS-HCl (pH 8.0), 500 mM NaCl, 15% glycerol and 20 mM imidazole, supplemented with protease inhibitors cocktail (Roche Diagnostics), sonicated and centrifuged 1 h at 14000×g. The supernatant was incubated with Ni-NTA beads for 2 hours with gentle rotation at 4°C. Fractions containing His-tag fusion proteins eluted with 250 mM imidazole were pooled and dialyzed overnight in buffer containing 20 mM TRIS-HCl (pH 8.0), 500 mM NaCl, 15% glycerol and 5 mM DTT. Samples were concentrated and loaded onto a Superdex 75 gel filtration column equilibrated in 20 mM TRIS-HCl (pH 8.0), 150 mM NaCl, 10% glycerol and 5 mM DTT buffer. Finally, fractions containing JAK2 proteins were concentrated and analyzed by Western Blot using anti-pTyr1007/1008 JAK2 antibodies and anti-His antibody diluted 1∶1000 in TBS buffer, followed by secondary biotinylated anti-Mouse or anti-Rabbit antibodies diluted 1∶3000 in TBS buffer and streptavidin-biotinylated horseradish peroxidase complex antibody diluted 1∶5000 in TBS buffer.

### PamChip® peptide microarrays for substrate identification and determination of catalytic efficiency

PamChip® Tyrosine kinase microarrays containing 144 peptides (13–15 amino-acids long) derived from putative tyrosine-phosphorylation sites in human proteins were used for substrate identification and spotted at a 1000 µM concentration as described elsewhere [24]. Peptides were named based on protein identities and amino acid position numbers, as described in UniProt Knowledgebase. A pre-tyrosine-phosphorylated peptide and a peptide lacking tyrosine residues were used as a control for antibody recognition.

For substrate preference 24 peptides were selected and printed on a custom PamChip® microarray containing peptide concentration series at 100, 300, 400, 600, 750 and 1000 µM, as confirmed by fluorescence intensity measurements after staining of arrays with Sypro Ruby Protein Blot Stain (Molecular Probes, Invitrogen). Selection of peptides was based on two main criteria: firstly, peptides should give signals with at least one of the proteins tested; and, secondly, peptides should belong to proteins reported to interact with JAK2 (according to the human protein reference database). Incubations were performed in triplicates with 2 pmoles per reaction JH1-JH2WT, 0.4 pmoles per reaction JH1-JH2V617F, 0.04 pmoles JAK2 JH1 and 1.2 pmoles JAK3 JH1 in a final reaction volume of 25 µl. Reactions were incubated with 400 µM final ATP concentration.

Incubations and dynamic readings of the peptide microarrays were performed at 30°C on a PamStation 96 instrument (PamGene International BV) that allows simultaneous incubation of 96 arrays. Prior to incubation with the kinase buffer, arrays were blocked with 2% BSA (w/v, Fraction V, Calbiochem) in water for 30 cycles and washed three times with PK assay buffer (50 mM Tris–HCl [pH 7.5], 10 mM MgCl2, 1 mM EGTA, 2 mM DTT, and 0.01% Brij-35). The kinase reactions (1× PK kinase buffer, JAK proteins at indicated concentration, 1× BSA, 12.5 µg/ml fluorescein-labeled PY20 antibody and 400 µM ATP) were incubated for 60 cycles of pumping up and down through the pores of the microarrays at a rate of 2 cycles per minute. Arrays were imaged every second cycle by an integrated CCD-based optical system. A representation of the typical workflow is shown in Figure S1.

### Signal quantification and data analysis

Time series of images were analyzed and signal intensities were quantified by BioNavigator software. To determine the initial reaction rate (v), the signal minus background from the time series of each spot (Figure 1C) was fitted to an equation for exponential association y = y_0_+y_max_(1 – e^−kc^), where y value stands for the signal intensity at cycle of measurement, k is the reaction rate constant; and c is the cycle number when the image was recorded. The initial velocity of peptide phosphorylation (v) was determined at the second data point via v = y_max_ * k * e^−kc^. Signal intensities after 20 min of incubation were also determined and used for quality control of the initial velocities. Only v values were used for subsequent data interpretation. Initial velocities (v) for each peptide, determined from the time course of the reactions as described above, were expressed as percentage of activity of the JAK2 protein on 1000 µM STA5A_687_699. Rates were expressed per pmol of protein. These values were analyzed using Graphpad Prism 4.0 software. Data presented are the average of 3 technical replicates.

## Supporting Information

Figure S1 **Typical workflow diagram of a PamChip® peptide microarray experiment.** PamChip® arrays are spotted on a porous 3-D layer of metal oxide. The presence of pores increases the surface area and allows immobilization of a high concentration of peptide in each of the 144 spots. By using a PamStation, 96 samples on a 96 array plate can be effectively pumped up and down through the pores. Upon kinase and ATP addition phosphorylated peptides are detected by a fluorescein-labeled PY20 antibody. The physical properties of the material (translucent when wetted) allow real-time detection of fluorescent signals by a charge-coupled device (CCD) camera. Signal intensities in each spot and its background are obtained from each image by Bionavigator. This software calculates the signal minus background of each spot at each time point and fits initial reaction rates through the time series. Finally, initial reaction rates (average of triplicate incubations) are used in the calculation of kinetic parameters using specific Graphpad Prism 4.0 software.
(TIF)

Figure S2 **Relative activity on different peptides.** **A**. Comparison of K_m_ values for JAK2 JH1, JAK2 JH1-JH2WT and JH1-JH2V617F for EGFR_1190_1202 peptide. Relative activity was calculated in relation to the initial rates obtained for 1000 µM EGFR_1190_1202 for each protein. Values are the average of 3 v-values. **B**. Comparison of K_m_ values for JAK2 JH1, JAK2 JH1-JH2WT and JH1-JH2V617F for STA5A_687_699 peptide. Relative activity was calculated in relation to the initial rates obtained for 1000 µM STA5A_687_699 for each protein. Values are the average of 3 v-values.
(EPS)
