# Supplementary figures and images for: Analysis of Jak2 Catalytic Function by Peptide Microarrays: The Role of the JH2 Domain and V617F Mutation
Source: PLoS One. 2011 Apr 18;6(4):e18522. doi: 10.1371/journal.pone.0018522 (PMC3078918; doi:10.1371/journal.pone.0018522)

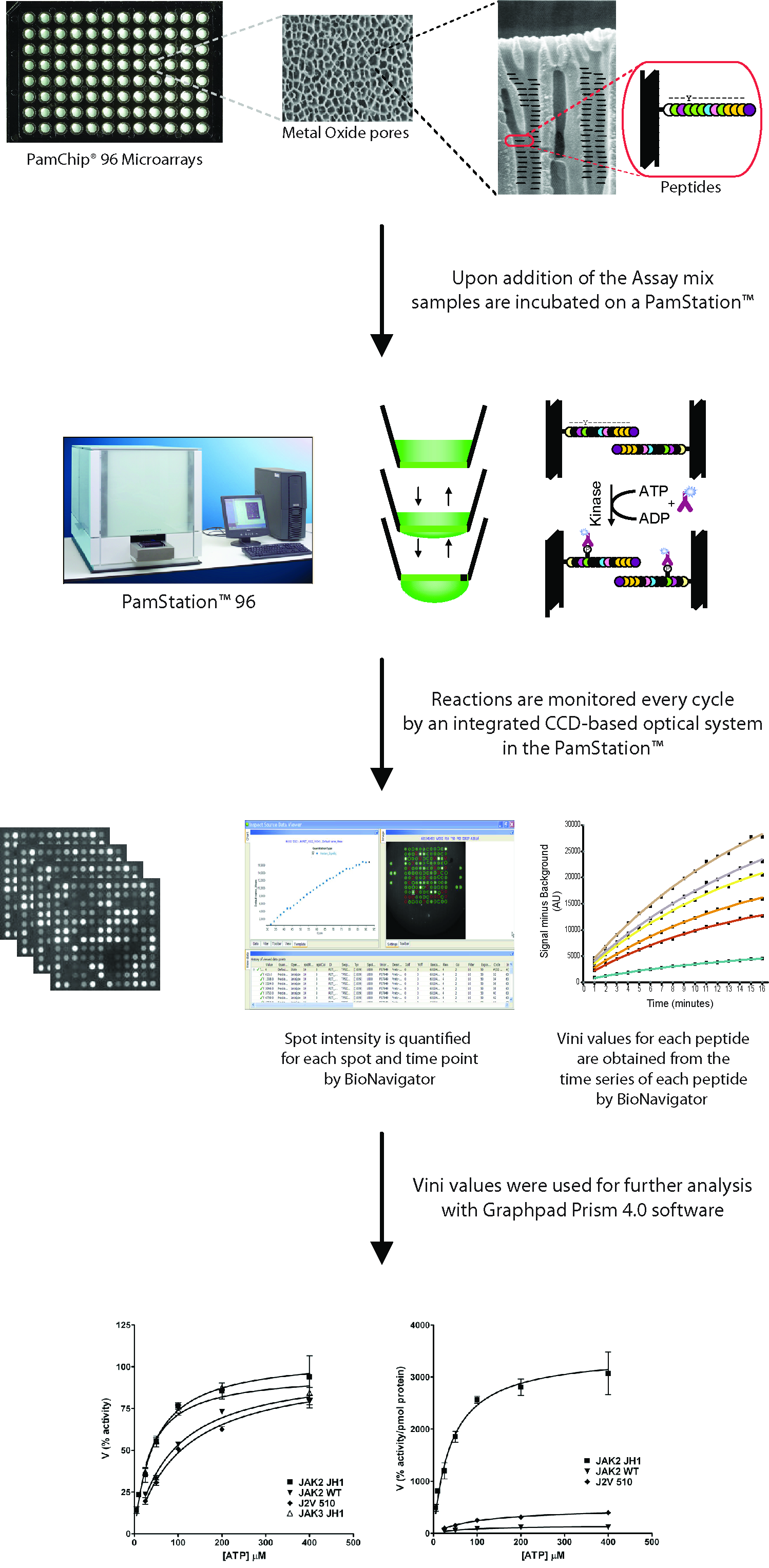

Supplement: Figure S1 — Typical workflow diagram of a PamChip® peptide microarray experiment. PamChip® arrays are spotted on a porous 3-D layer of metal oxide. The presence of pores increases the surface area and allows immobilization of a high concentration of peptide in each of the 144 spots. By using a PamStation, 96 samples on a 96 array plate can be effectively pumped up and down through the pores. Upon kinase and ATP addition phosphorylated peptides are detected by a fluorescein-labeled PY20 antibody. The physical properties of the material (translucent when wetted) allow real-time detection of fluorescent signals by a charge-coupled device (CCD) camera. Signal intensities in each spot and its background are obtained from each image by Bionavigator. This software calculates the signal minus background of each spot at each time point and fits initial reaction rates through the time series. Finally, initial reaction rates (average of triplicate incubations) are used in the calculation of kinetic parameters using specific Graphpad Prism 4.0 software. (TIF) [file pone.0018522.s001.tif]
